# Lipid Catabolism in Starved Yak Is Inhibited by Intravenous Infusion of β-Hydroxybutyrate

**DOI:** 10.3390/ani10010136

**Published:** 2020-01-15

**Authors:** Huawei Zou, Rui Hu, Xianwen Dong, Ali Mujtaba Shah, Zhisheng Wang, Jian Ma, Quanhui Peng, Bai Xue, Lizhi Wang, Xiangfei Zhang, Shaoyu Zeng, Xueying Wang, Junhua Shi, Fengpeng Li

**Affiliations:** 1 “Low Carbon Breeding Cattle and Safety Production” University Key Laboratory of Sichuan Province, Animal Nutrition Institute, Sichuan Agricultural University, Chengdu 61130, China; zhwbabarla@126.com (H.Z.); ruitianhu@yeah.net (R.H.); dxwcqxky@163.com (X.D.); alimujtabashah@sbbuvas.edu.pk (A.M.S.); Crazyma0411@163.com (J.M.); pengquanhui@126.com (Q.P.); xuebai68@163.com (B.X.); wanglizhi08@aliyun.com (L.W.); zxfsicau@foxmail.com (X.Z.); ifraanwar151@gmail.com (S.Z.); wangxuey_91@163.com (X.W.); mujtaba43@gmail.com (J.S.); fengpli@126.com (F.L.); 2 Department of Livestock Production, Shaheed Benazir Bhutto University of Veterinary and Animal Sciences, Sakrand 67210, Pakistan

**Keywords:** yak, starvation, β-hydroxybutyrate, lipid metabolism, GPR109A

## Abstract

**Simple Summary:**

Yak, which is the predominant and semi-domesticated livestock on the Qinghai-Tibet Plateau, suffers severe starvation and body weight reduction in the cold season because of the harsh highland environment. Lipids are important energy sources to starvation animals. β-hydroxybutyrate (BHBA) that is derived from lipid decomposition as the primary ketone body is with the function not only to provide energy for animals as energy materials, but also regulate lipid metabolism as signaling molecular. However, the effects of starvation and BHBA on lipid metabolism and its mechanism are still unclear for ruminant animals. Herein, we investigated the effects of starvation and intravenous infusion of BHBA solution on Yak growth, serum biochemistry, hormones, subcutaneous adipocyte morphology, fatty acid composition, activity of enzymes related to lipid metabolism, and signal pathway. The results showed that starvation promoted lipid catabolism and BHBA infusion up-regulated the mRNA expression of receptor GPR109A in subcutaneous adipose tissue, inhibited the Cyclic adenosine monophosphate(cAMP)/Protein kinase A (PKA)/cAMP-responsive element binding protein (CREB) signaling pathway, and inhibited lipolysis. Our study was beneficial for enriching the nutrition regulation theory of yaks and improving their growth potential.

**Abstract:**

Lipid is the chief energy source for starved animals. β-hydroxybutyrate (BHBA) is the main ketone body produced by lipid decomposition. In Chinese hamster ovary (CHO) cell experiment, it was found that BHBA could be used not only as an energy substance, but also as a ligand of GPR109A for regulating lipid metabolism. However, whether BHBA can regulate lipid metabolism of yaks, and its effective concentration and signal pathway are not clear. This study investigated the effects and mechanism of starvation and BHBA on the lipid metabolism of yak. Eighteen male Jiulong yaks were selected and then randomly divided into three groups: normal feeding group (NG), starvation group (SG), and starvation with BHBA infusion group (SBG). The yaks in the NG group were freely fed during the trial, while the yaks in the SG and SBG groups fasted; from 7th to 9th days of the experiment, the NG and SG were infused continuous with 0.9% normal saline and SBG was infused 1.7 mmol/L BHBA solution respectively. The blood samples were collected on the 0th, 1st, 3rd, 5th, 7th, and 9th day of experiment. The subcutaneous adipose tissue of all the yaks in this study were taken from live bodies after infusion. Serum glucose, lipid metabolites, hormone concentrations, and mRNA and protein expressions of key factors of lipid metabolism and signaling pathway in subcutaneous adipose tissue were measured. The results showed that, as compared with NG, starvation significantly reduced the body weight of yak in SG, and significantly increased the concentration of BHBA in serum and the mRNA expression of PKA and CREB1 in subcutaneous adipose tissue, while the mRNA expression of MEK, PKC, ERK1/2, the area of adipocytes, and the proportion of saturated fatty acid were decreased. Whereas, further increase of BHBA concentration through infusion promoted the mRNA expression of GPR109A receptor in the subcutaneous adipose tissue of SBG, inhibited the mRNA expression of AC and PKA, and decreased the phosphorylation protein abundance of CREB1, and significantly increased the diameter and area of adipocytes. These findings suggest that starvation led to enhanced lipid catabolism in yaks. An increasing BHBA concentration could increase the mRNA expression of GPR109A receptor in subcutaneous adipose tissue and inhibit the cAMP/PKA/CREB signaling pathway and lipid decomposition.

## 1. Introduction

Yak (*Bos grunniens*) is the unique bovine that has evolved to adapt to the harsh environment of Qinghai-Tibetan Plateau over 3500 m above sea level. Currently, most of the yaks are still in the semi-wild status raised by the natural grazing system, because of the harsh climate conditions. The Qinghai-Tibet Plateau climate is sharp frost in the long-term cold season from October to May (average temperature 5–15 °C) [1,2]. The plateau grassland has a long-term withered period (October to May of the next year) every year when forage is extreme scarce; thus, yaks inevitably suffered from serious starvation in the seasonal cycles, which caused substantial economic losses. Therefore, investigating the regulatory mechanisms of nutritional metabolism under starvation is meaningful for yak farming on the Qinghai-Tibetan Plateau.

The deposition of adipose tissues is the essential energy storages in the body of yaks for survival under prolonged starvation. As starvation progressed, animals altered the metabolic strategy from utilizing carbohydrates to triglycerides to provide energy for the function of the essential organs, such as heart, brain, and muscle, to maintain energy homeostasis of the body [1,3]. The deposition of the triacylglycerol in the fat tissue was mobilized to produce non-esterified fatty acid (NEFA) and glycerol. The NEFA is catabolized through β-oxidation in peripheral tissues and it produces acetyl-coenzyme A that can be converted to ketone bodies through the ketogenesis pathway in the hepatic mitochondrial matrix. Firstly, the acetyl-coenzyme A that is derived from β-oxidation is condensed to acetoacetic acid (ACAC) under the action of 3-hydroxy-3-methylglutaryl-Coenzyme A synthase 2 (HMGCS2) and 3-Hydroxy-3-methylglutary-CoA lyase (HMGCL). Afterwards, ACAC can be spontaneously converted to acetone, or to BHBA under the action of BHB dehydrogenase (BDH1). As a more stable form, BHBA is exported to the blood for use in extrahepatic tissues. The β-hydroxybutyrate (BHBA) is the predominant component of ketone body and it acts as a crucial alternative energy fuel for extrahepatic tissues during nutrient deprivation. Our previous study found that the concentrations of NEFA and BHBA in yak serum significantly increased after starvation [1]. However, ketosis can be caused by ketone bodies that are produced by the liver that exceed its metabolic capacity [4]. Whereas, after the long-term severe starvation, the deposition of adipose tissue is excessively mobilized and exhausted, and life is endangered, and possibly premature death occurs because of metabolic disorders. Thus, the optimal strategy and fundamental principle for prolonging life during long-term starvation might be the reductions in metabolic rate and energy expenditure to meet the minimum requirement of life [5].

The emerging studies have found that BHBA is not only a metabolic intermediate of physiological fuel, but also has a signaling function for regulating the energy expenditure and homeostasis during nutritional deprivation. For example, BHBA inhibited the adipose lipolysis in mouse and human fat tissue [6,7], decreased sympathetic activity [8], and also decreased the catecholamine-induced thermogenesis in the adipocytes of rats [9], which is involved in reducing the overall metabolic rate and saving energy expenditure. Therefore, BHBA is involved in a negative feedback loop to repress the excess production of NEFA and ketoacidosis, to prevent the hyperactivation of fat storage mobilization and metabolic dysfunction, which was considered as a vital metabolic mechanism to survive organisms during nutritional deprivation [7,10]. Taggart et al. (2005) [7] reported that the fatty acid-derived ketone body (d)-β-hydroxybutyrate specifically activates PUMA-G/HM74a at concentrations that were observed in serum during fasting. Racemic (dl)-β-hydroxybutyrate is more active. The half-maximal concentration (EC50) of racemic (dl)-β-hydroxybutyrate to stimulate HM74a was 0.7 mmol/L, which was higher than the BHBA concentration (0.39mmol/l) found in yak serum after seven days of starvation in our previous study [1]. Wang (2013) [11] reported that BHBA (2.5 mmol/L) bind to GPR109A and lead to the dissociation of heterotrimeric G protein complex into Gαi and βγ subunit, thereby inhibiting AC activity. Recently, GPR109A reported highly expressed in adipose tissue of cattle [12]. Kenez et al. (2014) [13] reported that GPR109A involved in inhibiting excessive lipomobilization in dairy cow during transition period and proved that GPR109A mediated the BHBA decreasing lipolysis in the adipose tissue explants of dairy cow. However, whether BHBA can activate GPR109A receptor in subcutaneous adipose tissues of yak, as well as its effective concentration and the signaling mechanisms of BHBA regulated lipid metabolism in the adipose tissue of starved yak, remain unclear. Therefore, the purpose of this study was to evaluate the molecular mechanism of lipid metabolism in yak adaption to starvation stress and the infusion of BHBA.

## 2. Materials and Methods

All of the experimental procedures that were used in this research were in accordance with the ARRIVE guidelines and the Regulation on the Administration of Laboratory Animals (2017, China State Council). The Institutional Animal Care and Use Committee of Sichuan Agricultural University approved these procedures.

### 2.1. Experimental Animals, Design and Diet

A total of eighteen three-year-old healthy male yaks (249.4 ± 12.53 kg, mean ± SEM) were randomly selected and then divided into three groups with six yaks in each group. The three groups were treated as normal feeding group (NG), starvation group (SG), and starvation with BHBA infusion group (SBG). The yaks were fed in a single pen, and water was provided *ad libitum*. All of the yaks were tethered. After 14-days of the adjustment period, yaks of the starved and BHBA infusion groups were starved without fed any diet for nine days (day 0–9). The nine-day starvation time was determined by our previous work to ensure that yaks were in the state of negative energy balance and did not reach a critical stage of life after starved [1,14]. Yaks of the NG fed with the experimental diet [1] *ad libitum* throughout the trial period. All of the yaks received a continuous 48-h intravenous infusion from 9:00 a.m. on the 7th day to 9:00 on the 9th day. The yaks in the NG and SG groups were infused with 0.9% saline, while the SBG was infused with BHBA solution (1.7 mmol/L).

### 2.2. BHBA Infusion

The BHBA solution was prepared in accordance with the previous study [15]. Briefly, the DL-BHBA acid sodium salt (bought from Sigma company, St. Louis, MO, USA) was dissolved in ultrapure water to the concentration of 1.7 mmol/L. Subsequently, the pH value of the solution was adjusted to 7.4 while using HCl and autoclaved at 131 °C and 100 kPa for 50 min. The solution was stored at 4 °C immediately after being filtered through the 0.2-μm filter.

The indwelling intravenous catheters (Shanghai Pudong Jinhuan Medical Supplies Co., LTD, Shanghai, China) were fitted on both of the ear veins of each yak on day 7. The infusion through the left-side catheters of yaks by a peristaltic pump (Shenzhen Haoke Medical Instrument Co. LTD, Shenzhen, China). The initial infusion dose was calculated based on the body weight of yaks (8.5 μmol/kg per min). During the first 2 h after infusion, the blood samples were collected through the right-side catheters every 15 min and then determined the BHBA concentration immediately by using blood ketone meter (Hebei Dizhun biotechnology Co. LTD, Shijiazhuang, China). The BHBA infusion rate was instantly adjusted to maintain the blood BHBA content between 1.5 to 2.0 mmol/L, so as to avoid ketosis that is caused by too high BHBA concentration. The 0.9% saline solution was infused to the yaks of control and starvation group with the same infusion time and rate.

### 2.3. Sample Collection and Analysis

#### 2.3.1. Sample Collection

Body weight was weighed at 0900 h on day 0, 7, and 9. Jugular blood samples were collected at 0900 h on the 0th, 1st, 3rd, 5th, 7th day of this study and 0.5th, 1st, 2nd, 4th, 8th, 12th, 24th, and 48th hour after infusion. Subsequently, the serums were separated and stored at −20 °C. Subcutaneous fat tissues were collected in the region of tailhead by biopsy on day 9 after infusion, according to the method that was described by Locher et al. [16]. Subsequently, adipose tissue biopsies were rinsed while using sterile saline solution and then divided into two parts, one part (6 g) was immediately frozen by liquid nitrogen and then stored at −80 °C for further analysis, the other part was fixed in 4% paraformaldehyde overnight for morphological analysis.

#### 2.3.2. Blood Serum Biochemical Indices and Hormones

The concentration of glucose (GLU), BHBA, total triglyceride (TG), non-esterified fatty acid (NEFA), total cholesterol (TC), total protein (TP), urea nitrogen (BUN) in serum were analyzed by Hitachi Automatic Analyzer 3100 (Hitachi, Tokyo, Japan). The concentration of insulin (INS), glucagon (GC), growth hormone (GH), and insulin-like growth factor-1 (IGF-1) in serum were detected by ELISA kits for bovine (Kamaishu Biotechnology Co., LTD, Shanghai, China) via microplate spectrophotometer (SpectraMax 190, Molecular Devices Corp., Sunnyvale, CA, USA). The inter-assay and intra-assay coefficient of variation (CV) of these serum hormones assays were below 15% and the lowest detection limit was 0.1 mIU/L (for INS), 1.0 pg/mL (for GC), 0.1 ng/mL (for GH), and 1.0 ng/mL (for IGF-1).

#### 2.3.3. Key Enzyme Activity of Fat Metabolism

The activity of diacylglycerol *O*-acyltransferase-1 (DGAT-1), fatty acid synthase (FAS), acetyl CoA carboxylase (ACC), acyl-coenzyme A oxidase (ACOX), and hormone-sensitive esterase (HSL) in subcutaneous adipose tissue were determined by ELISA kit for bovine (Kamaishu Biotechnology Co., LTD, Shanghai, China) via the same instrument described above. The inter-assay and intra-assay CV of these serum hormones assays were below 15% and the lowest detection limit was 0.1 U/mL (for DGAT-1), 10 U/mL (for FAS), 1.0 U/L (for ACC), 1.0 U/mL (for ACOX), and 10 U/L (for HSL).

#### 2.3.4. Fatty Acid Composition

The fatty acids methyl esters were derivatized from subcutaneous fat samples according to the GB/T 17376-2008 standard. Briefly, approximately 250 mg of subcutaneous fat tissues were taken and added 6 mL of KOH-CH3OH solution (0.5mol/L). After 1 min vortex oscillation, these samples were heated in the water bath at 90 °C until the oil drops completely disappeared. After cooling for 3 min, the samples were added 2 mL of BF3-CH3OH solution (w = 10%) and then methylated in water bath at 80 °C for 2 min. After cooling to room temperature, samples were added 1 mL of n-hexane and 5 mL of saturated NaCl solution, then mixed and centrifuged at 2000 rpm for 5 min at room temperature. Finally, the *N*-hexane layer (not less than 500 μL) was absorbed for gas chromatography (Varian 3800 GC) detection with reference to Noci et al. (2005) [17].

#### 2.3.5. Adipose Tissue Histomorphology

The subcutaneous adipose tissues were placed into 4% paraformaldehyde overnight and then dehydrated and embedded in the paraffin and cut into three sections with 5 μm thick and stained with Hematoxylin and Eosin (H&E). Five images were randomly captured in each stained section while using a light microscope with a digital color camera at 200× magnification. Adipocyte diameter (mm) and areas (mm^2^) of 20 adipocytes from each image were measured by using Image-Pro Plus 6.0 software (Media Cybernetics Inc., Bethesda, MD, USA) [18].

#### 2.3.6. qPCR

The total RNA was extracted while using Trizol reagent (Takara, Dalian, China) and then DNase I (Invitrogen, Carlsbad, CA, USA) was used for DNA digestion. RNA concentration was detected by using nanodrop (Takara, Dalian, China). RNA quality was determined through the Agilent 2100 bioanalyzer (Agilent Technologies, Santa Clara, CA, USA), all of RNA samples with RNA integrity number value >7.0 were used. The cDNA synthesis was performed using RT-PCR. Briefly, RNA was diluted to 100 ng/μL, then 2 μL of RNA, 1 μL of Random Primers (Roche, Basel, Switzerland) and 9 μL of RNase-free water was mixed and then incubated at 65 °C for 5 min in an Eppendorf Mastercycler Gradient (Fisher Scientific, Waltham, MA, USA). Afterwards, 4 μL of 5× First-Strand buffer (Fermentas, Pittsburgh, PA, USA), 1 μL of oligo dT18, 2 μL of 10 mM deoxynucleotide 5′-triphosphate mix (Invitrogen), 0.25 μL of reverse aid RT (Fermentas), 0.125 μL of RNase inhibitor (Fermentas), and 1.625 μL of RNase-free water were added and incubated with following temperature program: 25 °C for 5 min, 42 °C for 60 min, and 70 °C for 5 min. The cDNA was then stored at −20 °C. According to the primer sequence of the target gene queried in GenBank, primer 5.0 software was used to design the primer. Table 1 reports the primer list. Subsequently, q-PCR was performed by using the SYBR Green Kit (Takara, Dalian, China). The relative expressions were calculated while using 2^−ΔΔCt^ method, and the housekeeping genes were chosen according to the previous study [19]. All of the detected genes were with amplification efficiencies between 90–100%.

#### 2.3.7. Western Blot

The protein samples with four replicates per treatment were used for Western blot analysis, as previously reported [20]. In brief, 20 μg proteins of each sample were diluted while using 4× Laemmli sample buffer (no. 161-0474; Bio-Rad, Hercules, CA, USA) with ratio of 3:1, and then boiled at 100 °C for 10 min in a waterbath. Subsequently, the samples were then added into 10% SDS/PAGE at 120 V for approximately 120 min following with electrotransfering onto a PVDF membrane via semi-dry electrophoretic transfer cell (no. 170-3940; Bio-Rad, Hercules, CA, USA). The polyvinylidene fluoride (PVDF) membranes were blocked with 5% skim milk for 1h at room temperature and then incubated overnight at 4 °C with the primary antibodies (Table 2), followed by six five-minute washes in Tris-buffered saline-Tween buffer. Subsequently, the incubation of secondary antibody was performed at room temperature for 1 h with the same wash procedure. The visualization of PVDF membranes was performed by Clarity Western ECL substrate (no. 170-5060; Bio-Rad, Hercules, CA, USA) and ChemiDoc MP system (Bio-Rad). The quantification of band intensity was with Image Lab software (version 3.0, Bio-Rad, Hercules, CA, USA). Phosphorylation state of protein was calculated by phosphorylated protein/total protein (arbitrary units). GAPDH was used as the internal reference protein.

### 2.4. Statistical Analyses

All of the real-time PCR and Western blot data with non-normal distribution were log2 transformed before statistical analysis. One-way ANOVA analyzed the differences of data related to growth performance, serum parameters, serum hormones, fatty acid composition, adipocytes size, mRNA, and protein expressions between groups after Duncan’s post hoc testing in SPSS 20.0 (SPSS Inc., Chicago, IL, USA). The serum metabolite concentrations, such as GLU, BHBA, TG, TC, and NEFA, at 0–7 days of the experiment were also analyzed while using the SPSS Mixed models, and take treatment and days as factors. Statistical significance was considered at *p* ≤ 0.05.

## 3. Results

### 3.1. Effect of Starvation and BHBA Infusion on Body Weight of Yaks

**Figure 1:**
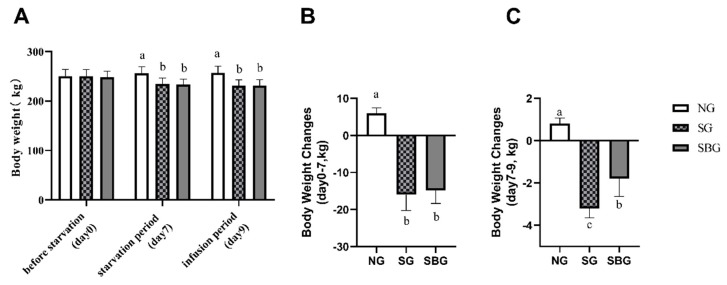
Effect of starvation and β-hydroxybutyrate (BHBA) infusion on yak body weight. (**A**) The body weight of yaks in day 0, day 7, and day 9. (**B**) the change of the body weight in the starvation period (day 0–7). (**C**) the change of the body weight in the infusion period (day 7–9). NG = Normal feeding group; SG = Starvation group; SBG = Starvation with BHBA infusion. a, b, c: Different superscript letters represent significant differences (*p* < 0.05).

The body weight (BW) was significantly influenced by the treatment of starvation and post-starvation with BHBA infusion, as shown in Figure 1. A similar BW was found in the three groups before starvation. On the 7th day of the experiment, as compared with NG, a significant decrease of BW was detected in group SG and SBG due to starvation (*p* < 0.05). On the ninth day of the experiment, when compared with SG, although the body weight of yaks with BHBA infusion after starvation did not significantly increase, the weight loss of yaks was reduced from day 7 to day 9 (*p* < 0.05).

### 3.2. Effect of Starvation and BHBA Infusion on Serum Biochemical Indicators

**Figure 2:**
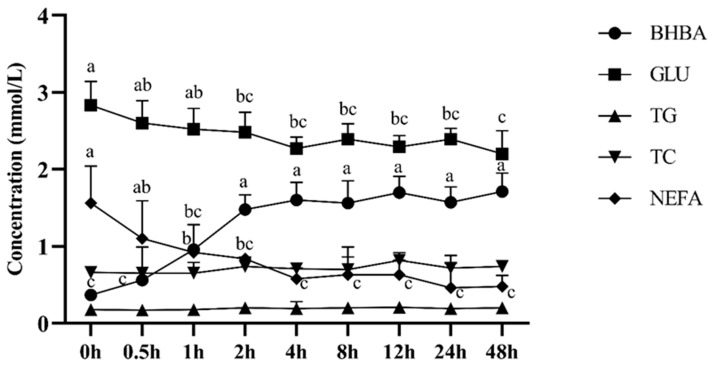
Dynamic changes of serum metabolites in 48 h of BHBA infusion, BHBA = β-hydroxybutyrate; GLU = Glucose; TG = Triglyceride; TC = Total cholesterol; NEFA = Non-esterified fatty acid. a, b, c: Different superscript letters represent significant differences (*p* < 0.05).

Figure 2 depicts the dynamic changes of serum metabolites in 48 h of BHBA infusion. The serum BHBA concentration of starved yak was significantly increased in two hours with BHBA infusion (*p* < 0.05). BHBA infusion led the lower (*p* < 0.05) concentration of GLU and NEFA at two or one hours, respectively. The infusion of BHBA had no significant effect on serum TC and TG concentrations.

**Figure 3:**
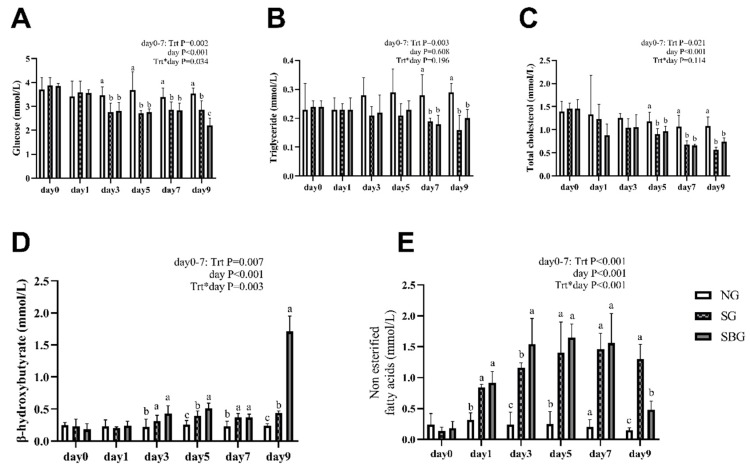
Effects of BHBA infusion on serum metabolites concentration of yak (mmol/L). Note: Data are shown as means ± SD. (**A**) the concentration of glucose in serum. (**B**) the concentration of triglyceride in serum. (**C**) the concentration of total cholesterol in serum. (**D**) the concentration of BHBA in serum. (**E**) the concentration of NEFA in serum. NG = Normal feeding group; SG = Starvation group; SBG = Starvation with BHBA infusion. a, b, c: Different superscript letters represent significant differences (*p* < 0.05).

Figure 3 reports the effect of BHBA infusion on serum metabolites concentration of yak. The results on the seventh day of the experiment showed that the concentrations of GLU, TG, and TC in SG and SBG were decreased by starvation (*p* < 0.05), while the concentrations of BHBA and NEFA were increased (*p* < 0.05). In SG and SBG groups, the concentration of glucose decreased significantly (*p* < 0.05) from the third day of starvation and then maintained a low and stable level, while the concentration of NEFA and BHBA significantly decreased on the first and third days, respectively (*p* < 0.05). TG might be a stable index in serum. A significant decreasing of TG was observed until day 7 after starvation (*p* < 0.05). From day 7 to day 9, starvation with BHBA infusion increased the serum BHBA concentration (*p* < 0.05), but further decreased the serum GLU concentration (*p* < 0.05). An increasing of NEFA, led by starvation, was inhibited (*p* < 0.05), due to BHBA infusion. Although BHBA infusion could inhibit the further decreasing of TG and TC, with no significant influence.

### 3.3. Effect of Starvation and BHBA Infusion on Serum Hormones

**Figure 4:**
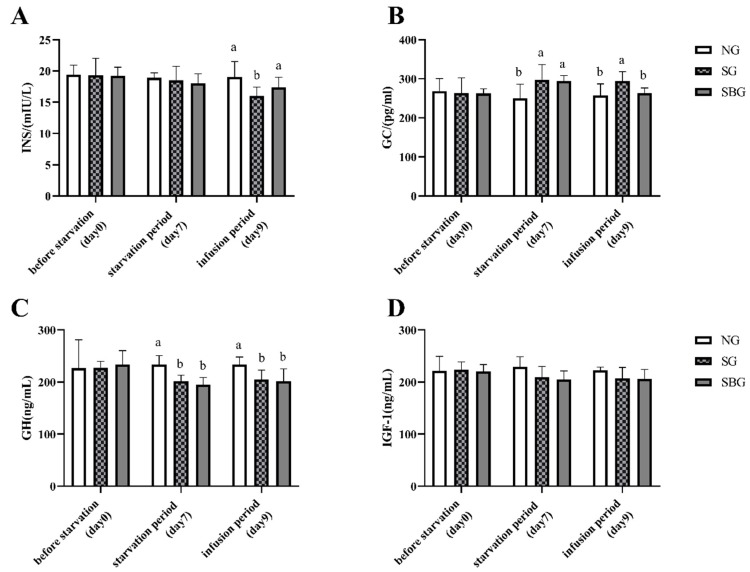
Effects of BHBA infusion on hormones in the serum of yak. NG = Normal feeding group; SG = Starvation group; SBG = Starvation with BHBA infusion. (**A**) The concentration of insulin in yak serum of before starvation period (day 0), starvation period (day 7), and infusion period (day 9). (**B**) The concentration of glucagon in yak serum of before starvation period (day 0), starvation period (day 7) and infusion period (day 9). (**C**) The concentration of growth hormone in yak serum of before starvation period (day 0), starvation period (day 7) and infusion period (day 9). (**D**) The concentration of insulin-like growth factor-1 in yak serum of before starvation period (day 0), starvation period (day 7), and infusion period (day 9). a, b, c: Different superscript letters represent significant differences (*p* > 0.05).

Figure 4 presents the results of the serum hormones of this study. On the 9th day of the experiment, the concentration of INS and GH in the serum of SG was lower than that of the NG (*p* < 0.05), and the concentration of GC was higher than that of the NG (*p* < 0.05). After starvation, the BHBA infusion reduced the serum GC concentration (*p* < 0.05), increased the concentration of INS (*p* < 0.05), but it had no significant effect on GH and IGF-I (*p* > 0.05).

### 3.4. Effect of Starvation and BHBA Infusion on Adipocytes Size

**Figure 5:**
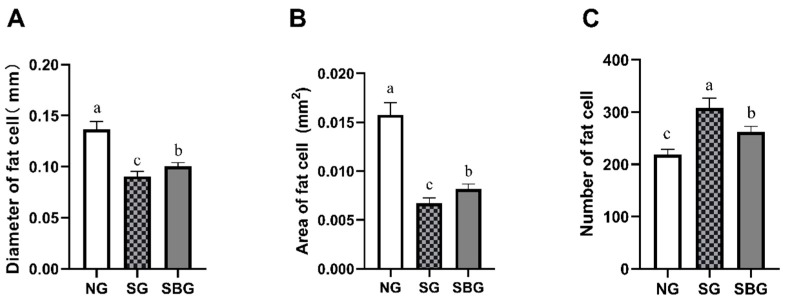
Effects of starvation and BHBA infusion on the size and quantity of subcutaneous fat cells in yak. (**A**) the diameter of adipocytes in yak subcutaneous adipose tissue. (**B**) the area of adipocytes in yak subcutaneous adipose tissue. (**C**) the number of adipocytes in yak subcutaneous adipose tissue. NG = Normal feeding group; SG = Starvation group; SBG = Starvation with BHBA infusion. a, b, c: Different superscript letters represent significant differences (*p* < 0.05).

Figure 5 shows the size and quantity of subcutaneous fat cells. Starvation and BHBA infusion decreased the diameter and area of fat cells (*p* < 0.05), but increased the number of fat cells (*p* < 0.05). When compared with SG, BHBA infusion led greater (*p* < 0.05) diameter and area of the fat cell, but lower (*p* < 0.05) number of fat cells.

### 3.5. Effect of Starvation and BHBA Infusion on Fatty Acid Composition

Table 3 depicts the effect of starvation and BHBA infusion on fatty acid composition in subcutaneous adipose tissue of yak. After nine days of starvation, as compared with NG, the proportion of total saturated fatty acid (SFA) in subcutaneous fat of SG decreased (*p* < 0.05), and the proportion of total monounsaturated fatty acid (MUFA) and total polyunsaturated fatty acid (PUFA) increased (*p* < 0.05). After 48 h of continuous infusion of BHBA, the proportion of SFA in SBG continued to decrease (*p* < 0.05), and the proportion of MUFA and PUFA continued to increase (*p* < 0.05). The proportion of stearic acid (c18:0) with the highest content of SFA in SG and SBG decreased by 17.72% and 24.09%, respectively, when compared with the NG (*p* < 0.05), while the proportion of oleic acid (c18:1n9c) with the highest content in MUFA increased by 43.49% and 55.18%, respectively (*p* < 0.05). The proportion of linoleic acid (c18:2n6c) in PUFA increased in SG and SBG (*p* < 0.05). Starvation reduced the proportion of arachidonic acid (C20:4n6) (*p* < 0.05), while BHBA infusion after starvation increased the proportion (*p* < 0.05), but it was still lower than that of the NG (*p* < 0.05).

### 3.6. Effect of Starvation and BHBA Infusion on the Activity of Enzyme and mRNA Expression of the Key Factor for Lipid Metabolism in the Adipose Tissues

**Figure 6:**
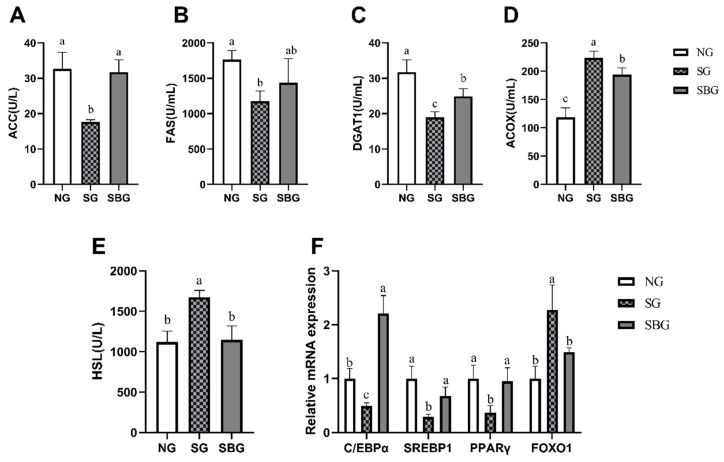
Effects of BHBA infusion on enzyme activity and mRNA expression of key factors for lipid metabolism in subcutaneous adipose tissue. (**A**) the activity of ACC in subcutaneous adipose tissue of yaks. (**B**) the activity of FAS in subcutaneous adipose tissue of yaks. (**C**) the activity of DGAT-1 in subcutaneous adipose tissue of yaks. (**D**) the activity of ACOX in subcutaneous adipose tissue of yaks. (**E**) the activity of HSL in subcutaneous adipose tissue of yaks. (**F**) the mRNA expression of key cytokines for lipid metabolism in subcutaneous adipose tissue of yaks. NG = Normal feeding group; SG = Starvation group; SBG = Starvation with BHBA infusion. a, b, c: Different superscript letters represent significant differences (*p* < 0.05).

The activity of ACC, FAS, and DGAT1 was lower (*p* < 0.05) in SG when compared with NG (Figure 6A–C), while the activity of ACOX and HSL was higher (*p* < 0.05) (Figure 5D–F). BHBA infusion led to higher (*p* < 0.05) activity of ACC and DGAT1 as compared with SG, but lower (*p* < 0.05) activity of ACOX and HSL. Starvation significantly decreased the expression of C/EBPα, SREBP1, and PPARα in subcutaneous adipose tissue of yak in SG (*p* < 0.05), and increased FOXO1 (*p* < 0.05) (Figure 3F). The infusion of BHBA after starvation up-regulated the expression of C/EBPα, SREBP1, and PPARα (*p* < 0.05), and down-regulated the FOXO1 (*p* < 0.05) when compared with SG.

### 3.7. Effect of Starvation and BHBA Infusion on the Signal Pathway of Lipid Metabolism

**Figure 7:**
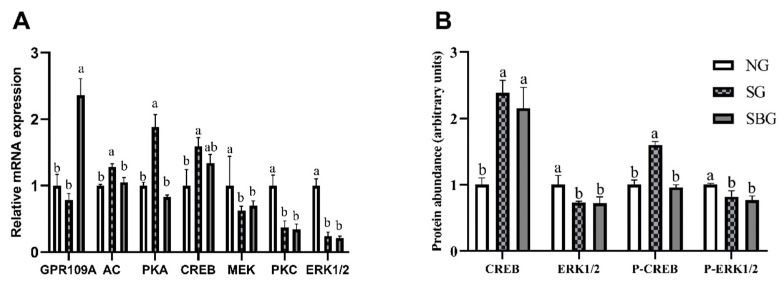
Effects of starvation and BHBA infusion on relative mRNA expression (**A**) and phosphorylation protein abundance (**B**) of key factors in the signal pathway of lipid metabolism. (**A**) the mRNA expression of key genes in BHBA mediated signaling pathway. (**B**) the expression and phosphorylation of key proteins in BHBA mediated signaling pathways. NG = Normal feeding group; SG = Starvation group; SBG = Starvation with BHBA infusion. a, b, c: Different superscript letters represent significant differences (*p* < 0.05).

mRNA expression (Figure 7A) of GPR109A and key factors in its downstream signaling pathway and phosphorylated protein expression (Figure 7B and Supplementary Figure S1) of CREB1 and ERK1/2 were detected to elucidate the regulation mechanism of starvation and BHBA on yak lipid metabolism (Figure 7). When compared with NG, starvation led greater (*p* < 0.05) mRNA expression of AC, PKA, and CREB1, lower (*p* < 0.05) PKC and ERK1/2 mRNA expression in SG group relative to NG group. When compared with SG, BHBA infusion increased mRNA expression of GPR109A (*p* < 0.05), and decreased mRNA expression of AC and PKA (*p* < 0.05) in the SBG group. Starvation increased the protein expression and phosphorylation level of CREB1 in subcutaneous adipose tissue of yaks in the SG group (*p* < 0.05), while decreased the protein expression and phosphorylation level of ERK1/2 (*p* < 0.05) when compared with NG group. After 48 h of BHBA infusion, the phosphorylation level of CREB1 protein in subcutaneous adipose tissue of yaks in SBG group was significantly lower than that in SG group (*p* < 0.05), while the effect on ERK1/2 protein expression and phosphorylation level was not significant (*p* < 0.05).

## 4. Discussions

### 4.1. Effects of Starvation and BHBA Infusion on Body Weight, Serum Biochemical Indicators, and Hormones

The weight loss is the most common response to starvation stress in animals, which is caused by the mobilization of body fat and other body storage [10]. Yu et al. [14] reported that the body weight of yaks decreased by 9.84% after nine days of starvation. The results of this experiment found that the body weight of the yak in SG decreased by 7.68% after nine days of starvation. Additionally, the results of this experiment showed that BHBA infusion significantly alleviated body weight reduction of starved yaks, which indicated that BHBA potentially decreased fat tissue depot mobilized of yaks under nutritional deprivation. This result suggested that exogenous BHBA might contribute to the survival of yaks under starvation.

Glucose is the primary energy source of animals at normal state. Brain, myocardium, and other tissues must rely on glucoses for energy supply. Therefore, it plays an essential role in maintaining the life activities of animals, and the body’s blood glucose must be kept stable. Under the condition of starvation, the insufficient intake of exogenous nutrients will lead to a decrease in blood glucose in the body [10]. Animals can use the catabolites of lipid to maintain the concentration of blood glucose stability through hepatic gluconeogenesis [21]. The previous studies of other animals have also found this phenomenon of blood glucose level, because the metabolic strategy transformed from carbohydrate to triglyceride to keep the blood glucose level stable [22]. In this study, the serum glucose level dramatically decreased from day 0 to day 3 after starvation and then remained stable from day 3 to day 9 after starvation. During starvation, triglycerides that were deposited in the adipocyte rapidly mobilize to produce glycerin and NEFA, which results in a sharp increase in NEFA concentrations in the blood, which are then oxidized and decomposed to provide energy for skeletal muscle, liver, and kidney [14,23]. Blood NEFA concentration is the best indicator for predicting body fat loss [24]. In addition, NEFA oxidation in the liver produces amounts of ketone body, such as BHBA. In this study, the increased concentrations of NEFA and BHBA in this study suggested that yaks mobilized amounts of adipose tissue depots to provide energy after starvation. Hormones play an important role in this process. Hormones, such as insulin and glucagon, regulate energy metabolism in animals [25]. Long-time fasting can induce insulin resistance in peripheral tissues, thus promoting glucose release to provide energy for brain and other organs [26]. The concentration of insulin in the serum of dogs that were starved for two days significantly decreased [27]. Yu et al. [14] found that from the second day after starvation, the serum insulin in yaks continued to decrease, and maintained a low level and stability after the fifth day. In this experiment, the serum insulin concentration of yaks in SG group significantly decreased until the 9th day of starvation. The reason for this difference may be that Yu et al. [14] conducted the study on the Qinghai Tibet Plateau with an altitude above 3600 m. During the experiment, the lowest temperature reached −36 °C and yaks suffered more severe starvation stress and cold stress, resulting in more energy consumption. The glucagon concentration, in this experiment, was significantly increased and then remained stable in the following period, so as to promote the gluconeogenesis in the state of starvation and insulin resistance, maintain low and stable blood glucose level, and promote yak adaptation to starvation. GH and IGF-1 can promote cell proliferation and protein deposition, thus promoting animal development and regulating nutrient metabolism [28]. GH and IGF-1 synergistically regulate the growth of animals, and the growth rate of animals has a significant positive correlation with the concentrations of GH and IGF-1 in the body [29]. Kate et al. [30] found that nutritional restriction significantly reduced the gene expression of GHR1A and IGF-1 in the liver of cattle. Hu et al. [31] also found that GHRH, GH, and IGF-1 in serum of growth retardation yak caused by nutrient deficiency showed a significant positive correlation with body weight, and cysteamine (somatostatin inhibitor) could increase the concentration of GHRH, GH, and IGF-1 in serum of growth retardation yak, thus promoting growth. In this study, the serum concentrations of GH and IGF-1 in yak after starvation were decreased, which could reduce the growth and metabolism of yak and, thus, help to reduce energy consumption.

After BHBA infusion, the level of serum BHBA significantly increased and then maintained at 1.5–2.0 mmol/L, which did not induce clinical ketosis [32]. Interestingly, the concentration of GLU and NEFA in serum decreased after the BHBA concentration reached 1.5–2.0 mmol/L through intravenous infusion, which suggested that BHBA inhibited the excessive lipolysis in adipose depots. This might be because BHBA replaces glucose to provide energy to peripheral tissues, such as the brain [33], thereby reducing the body’s need for glucose. Previous studies have reported exogenous BHBA prohibited the gluconeogenesis and reduced blood glucose level [34]. Insulin can inhibit glucose production by inhibiting the key enzyme of gluconeogenesis [35]. In this study, it was found that, after BHBA infusion, the serum insulin concentrations of yaks in SBG group were significantly higher than that of yaks in the SG group. This suggests that BHBA might inhibit gluconeogenesis by promoting insulin secretion. Xu et al. [36] reported that BHBA promoted mRNA expression of pyruvate carboxylase, which is a rate-limited enzyme of the liver gluconeogenesis at low concentration (<1 mmol/L), while it inhibited mRNA expression of pyruvate carboxylase at high concentration. BHBA infusion also reduced the serum glucose levels in dairy cows [15]. The results showed that starvation led to increased lipolysis and increased serum NEFA and BHBA concentrations. However, in this state, serum BHBA concentration was lower than 1.0mmol/L, which did not inhibit gluconeogenesis, and serum GLU concentration remained low and stable. Serum BHBA concentration was further increased after intravenous BHBA infusion, which resulted in a further decrease of GLU concentration and NEFA concentration in the serum of starvation yaks, indicating that BHBA is a vital energy substitute of animal under starvation.

### 4.2. Effects of Starvation and BHBA Infusion on Lipid Metabolism in Adipose Tissue

The adipose tissues are composed of fat cells and are important energy storage sites in animals and regulate metabolism under the nutrient deprivation situation. The lipid deposition in adult animals is mainly due to the increase in the volume of fat cells, rather than the increase in the number of fat cells [37]. Therefore, diets that are high in fat and calories increased fat cell volume [38], while diets that are restricted reduced the fat cell volume [39]. The decreased adipocytic diameter and areas of SG directly proved the mobilization of adipose depots of yaks under starvation. In addition, an increase in the area of fat cells in the SBG group might result in a decrease in the number of fat cells that can be observed in the same size field of vision. The consumption of fatty acids in different animals under starvation stress has a certain sequence [40]. This study found that the proportion of SFA in adipose tissue of yaks decreased after starvation, while the proportion of MUFA and PUFA increased, which indicated that the SFA in adipose tissue was first utilized by yaks during starvation. However, Yaffe et al. [41] found that the SFA and MUFA contents in the myocardium of fasted mice decreased, while the PUFA increased. This difference might be due to the different release rate, absorption efficiency, and specific oxidation of fatty acids in different animals or different tissues of the same animal. Interestingly, the size of adipocytes increased after the BHBA concentration reached 1.5–2.0 mmol/L through intravenous infusion. The results showed that BHBA could inhibit the decomposition and promote the deposition of lipid, but the 48-h infusion of BHBA did not restore the fat tissue to the same level as that of normal yaks without starvation.

PPARγ was found to promote the conversion of 3T3 fibroblasts to adipocytes by increasing the expression of CCAAT enhancer-binding protein α (C/EBPα) [42]. The knockout of PPARγ and C/EBPα genes led to a loss of adipose tissue [43,44]. In the cell culture experiment of preadipocytes, it was found that the mRNA expression of SREBP1 significantly increased during the differentiation of preadipocytes [45]. After silencing SREBP1 gene, the differentiation ability of preadipocytes was lost [46]. Moreover, SREBP1 also regulates the gene expression of lipid synthases, such as FAS and ACC [47]. FOXO1 could down-regulate the expression of SREBP1c [48] and inhibit the function of PPARγ [49]. Fasting reduces the expression of SREBP1c and FAS genes in skeletal muscle and liver and increases the expression of FOXO1 genes [48]. The results also found that starvation significantly reduced the mRNA expressions of C/EBPα, SREBP1, and PPARα in the subcutaneous fat tissues of yaks, and significantly increased FOXO1, which indicated that the differentiation of fat cells in subcutaneous adipose tissue of yak was inhibited and the lipid synthesis decreased after starvation. BHBA infusion could increase the mRNA expressions of C/EBPα, SREBP1, and PPARα in starved yak inhibits the mRNA expressions of FOXO1 and promotes the differentiation of fat cells.

The results of this study showed that, after nine days of starvation, the activities of the key enzymes of lipid synthesis, such as ACC, FAS, and DGAT-1, in the subcutaneous fat tissues of yaks in SG decreased. While the activities of key enzymes for lipid decomposition, such as ACOX and HSL, significantly increased after starvation [50], which indicated that starvation resulted in decreased lipid anabolism and enhanced catabolism in yaks. In the present study, the results also showed that BHBA infusion could significantly increase the activity of ACC, and reduced the activities of HSL and ACOX of starved yaks and, thus, inhibited the excessive lipid decomposition that is caused by starvation. Kenez et al. [13] reported that BHBA could promote the phosphorylation of serine residues in HSL by activating GRP109A receptor in fat cells, thus inhibiting lipid decomposition. The GPR109A is one of the specific receptors of BHBA and niacin in the adipocyte surface of various animals. Taggert et al. [7] found that the fatty acid-derived ketone body (d)-β-hydroxybutyrate specifically activates PUMA-G/HM74a at concentrations that were observed in serum during fasting, and Racemic (dl)-β-OHB had some degree of activity on all receptors. When its concentration is 2 mmol/L, lipolysis can be inhibited by regulating PUMA-G, but it does not have a significant effect in mice with PUMA-G gene knockout; a low concentration of BHBA (0.2 mmol /L) could not activate PUMA-G and the regulatory impact on lipid decomposition disappeared. Previous studies have found that the downstream signaling pathways that are mediated by GPR109A mainly include cAMP/PKA/CREB and MEK/PKC/ ERK1/2 pathways. The activation of cAMP/PKA/CREB pathway by the BHBA receptor GPR109A in the adipose cell line culture of the mouse was found to decrease the concentration of the second messenger cAMP in cells, inhibit the downstream regulatory factor CREB of PKA, reduce the activity of HSL, and inhibit lipolysis [51,52]. Wang [11] found that BHBA (2.5 mmol/L) significantly reduced the content of cAMP and the phosphorylation level of CREB in dairy cow anterior pituitary cells. Other studies have found that BHBA can regulate the MEK/PKC/ ERK1/2 signaling pathway through GPR109A to reduce the synthesis and secretion of the growth hormone-releasing hormone (GHRH) in the hypothalamus cells, thus promoting lipid synthesis [53]. Our results showed that the mRNA expressions of AC, PKA, and CREB1 in the subcutaneous adipose tissue of yak were increased, and the MEK, PKC, ERK1/2, and GH decreased, which was conducive to promoting the decomposition of lipid in starvation yak and, thus, providing energy for the body. After 48 h of infusion of BHBA solution, the mRNA expression of GPR109A increased, thereby significantly reducing the mRNA expression of AC and significantly reducing the phosphorylation protein abundance of CREB1, thus inhibiting lipid breakdown and promoting lipid synthesis. After BHBA infusion, there was no significant effect on the mRNA expression of MEK, PKC, and ERK1/2, and there was no significant change in GH concentration in the serum of starved yaks, which indicated this pathway and GH secretion mediated by this pathway did not regulate that the regulation of BHBA on subcutaneous fat metabolism of starved yaks.

## 5. Conclusions

The results revealed the effects of starvation and BHBA infusion on lipid metabolism and signaling pathways in yaks. Starvation caused weight loss in yaks, activated the cAMP/PKA/CREB signaling pathway, inhibited the MEK/PKC/ERK1/2 signaling pathway, and promoted the lipid metabolism, and mainly decomposed SFA, to maintain low and stable serum glucose concentration in starved yaks. In addition, intravenous BHBA infusion increased the concentration of BHBA in serum and improved the mRNA expression of GPR109A receptor in subcutaneous adipose tissue in starved yaks. However, intravenous BHBA infusion inhibited the cAMP/PKA/CREB signaling pathway and reduced the lipolysis, and it had no significant effect on MEK/PKC/ERK1/2 signaling pathway in subcutaneous adipose tissue and GH concentration in serum of starved yaks.

## Figures and Tables

**Table 1:** Primers used for q-PCR.

Genes	Primes	Accession No.
β-actin	F:5’ CAA GCA GGA GTA CGA TGA GT 3’	XM_005887322.2
	R: 5’ TTG TCA AGA AAA AGG GTG TAA C 3’	
PPARγ	F:5’ AAG CCC TTT GGT GAC TTT ATG 3’	XM_005902845.2
	R: 5’ GCA GCA GGT TGT CTT GTA TGT 3’	
C/EBPα	F:5’ GGA CAT GTG TGA GCA TGA G 3’	XM_005895434.2
	R: 5’ CGG AGA GGA GCT GTT CTT 3’	
SREBP1	F:5’ AGT TGA ATA AAT CTG CCG TCT T 3’	XM_014477492.1
	R: 5’ GCT TCT GGT TGC TGT GCT 3’	
FOXO1	F:5’ CTT CAG CCA GAG CAG TAT TT 3’	XM_005900262.2
	R: 5’ CTT TTT CCA GTT CCT TCA TTC 3’	
GPR109A	F:5’ GAC CTG GCG TTT TAC ATC AC 3’	XM_005911836.2
	R: 5’ ATG GGC TGG AGA AGT AGT ACA C 3’	
AC	F:5’ CGT TTC TCT CAC TTG CCT TTA 3’	XM_005905132.2
	R: 5’ ATG ATG CCC GAT GAC TTT A 3’	
PKA	F:5’ TCC CTT CTC ACT TCA GTT CA 3’	XM_005887423.2
	R: 5’ TAA ATA GCA ATC CAG TCT GTT GT 3’	
CREB1	F:5’ GAA GCA GCA CGA GAG TGT C 3’	XM_005907033.1
	R: 5’ GTC CTT TTC CCA CCA TCA TA 3’	
MEK	F:5’ CCG CAG AGA GAG CAG ATT T 3’	XM_005893283.2
	R: 5’ GCT TCC CAA CCA CTT AGA TG 3’	
PKC	F:5’ CCG CAA GCA GTG TTC TAT G 3’	XM_014479298.1
	R: 5’ TCT AAC TTC AGG TCC CGA TAA A 3’	
ERK1/2	F:5’ GCT CCT GCT CTG CTT ATG A 3’	XM_005907349.2
	R: 5’ ATT GAT TCC GAT GAT GTT CTC 3’	

Note: PPARγ = Peroxisome proliferator-activated receptors γ; C/EBPα = CCAAT/enhance binding proteins α; SREBP1= Sterol element binding proteins 1; FOXO1 = Forkhead box protein O1; GPR109A= G protein-coupled receptor 109A; AC = Adenylyl cyclase; PKA = Protein kinase A; CREB1 = cAMP-responsive element binding protein-1; MEK = Mitogen-activated protein kinase; PKC = Protein kinase C; ERK1/2 = Extracellular signal-regulated kinase 1/2.

**Table 2:** Antibodies used for Western Bloting.

Antibody	Cat No.	Dilution Ratio	Source	Manufacturer
CREB1	D120486	1:2000	Rabbit	Sangon Biotech
ERK1/2	D151973	1:2000	Rabbit	Sangon Biotech
P-CREB1	D151216	1:2000	Rabbit	Sangon Biotech
P-ERK1/2	D155117	1:2000	Rabbit	Sangon Biotech

Note: CREB1 = cAMP-responsive element binding protein-1; ERK = Extracellular signal-regulated kinase 1/2; P-CREB1 = Phosphorylated cAMP-responsive element binding protein-1; P-ERK1/2 = Phosphorylated Extracellular signal-regulated kinase 1/2.

**Table 3:** Effect of BHBA infusion on fatty acid composition in subcutaneous adipose tissue of yak (%).

Fatty Acids	NG	SG	SBG
∑SFA	70.911 ± 1.798 ^a^	61.00 ± 3.16 ^b^	57.636 ± 2.354 ^c^
C14:0	1.452 ± 0.247 ^a^	1.309 ± 0.133 ^ab^	1.218 ± 0.131 ^b^
C15:0	0.563 ± 0.041 ^a^	0.536 ± 0.089 ^ab^	0.462 ± 0.087 ^b^
C16:0	17.743 ± 2.376	17.019 ± 1.334	16.545 ± 1.272
C17:0	1.862 ± 0.18	1.75 ± 0.061	1.763 ± 0.072
C18:0	47.397 ± 3.923 ^a^	39.00 ± 3.804 ^b^	35.98 ± 1.458 ^b^
C20:0	1.045 ± 0.213 ^a^	0.783 ± 0.081 ^b^	0.816 ± 0.089 ^b^
C21:0	0.109 ± 0.035 ^a^	0.061 ± 0.013 ^b^	0.081 ± 0.03 ^ab^
C22:0	0.396 ± 0.123 ^a^	0.226 ± 0.041 ^b^	0.282 ± 0.047 ^b^
C23:0	0.099 ± 0.006 ^b^	0.141 ± 0.021 ^a^	0.176 ± 0.044 ^a^
C24:0	0.078 ± 0.026 ^a^	0.044 ± 0.004 ^b^	0.053 ± 0.011 ^b^
∑MUFA	27.354 ± 1.682 ^b^	37.054 ± 3.104 ^a^	40.255 ± 5.907 ^a^
C14:1	0.037 ± 0.013 ^b^	0.071 ± 0.031 ^a^	0.073 ± 0.032 ^a^
C15:1	0.005 ± 0.001 ^b^	0.007 ± 0.001 ^a^	0.006 ± 0.00 ^b^
C16:1	1.789 ± 0.216 ^b^	2.254 ± 0.498 ^ab^	2.625 ± 0.571 ^a^
C17:1	0.194 ± 0.055 ^b^	0.291 ± 0.049 ^a^	0.34 ± 0.094 ^a^
C18:1n9t	2.804 ± 0.43 ^a^	1.757 ± 0.306 ^b^	1.719 ± 0.65 ^b^
C18:1n9c	22.487 ± 1.587 ^b^	32.266 ± 2.446 ^a^	34.896 ± 5.817 ^a^
C20:1n9	0.136 ± 0.031 ^b^	0.144 ± 0.019 ^b^	0.188 ± 0.015 ^a^
C22:1n9	0.344 ± 0.086	0.251 ± 0.052	0.301 ± 0.082
C24:1n9	0.02 ± 0.007 ^a^	0.012 ± 0.002 ^ab^	0.016 ± 0.004 ^b^
∑PUFA	1.735 ± 0.126 ^c^	1.946 ± 0.113 ^b^	2.109 ± 0.145 ^a^
C18:2n6t	0.019 ± 0.007 ^a^	0.009 ± 0.004 ^b^	0.01 ± 0.004 ^b^
C18:2n6c	1.31 ± 0.156 ^b^	1.553 ± 0.07 ^a^	1.682 ± 0.131 ^a^
C18:3n6	0.001 ± 0.00 ^b^	0.023 ± 0.001 ^a^	0.001 ± 0.000 ^b^
C18:3n3	0.154 ± 0.03	0.182 ± 0.053	0.188 ± 0.041
C20:2	0.014 ± 0.005 ^c^	0.031 ± 0.003 ^b^	0.036 ± 0.005 ^a^
C20:3n6	0.014 ± 0.002 ^b^	0.02 ± 0.003 ^a^	0.025 ± 0.004 ^a^
C20:3n3	0.018 ± 0.003 ^ab^	0.013 ± 0.002 ^b^	0.021 ± 0.008 ^a^
C20:4n6	0.153 ± 0.006 ^a^	0.053 ± 0.019 ^c^	0.083 ± 0.024 ^b^
C22:2	0.043 ± 0.015 ^a^	0.024 ± 0.003 ^b^	0.029 ± 0.004^b^
C20:5n3	0.009 ± 0.002 ^b^	0.011 ± 0.003 ^ab^	0.015 ± 0.004 ^a^
C22:6n3	0.029 ± 0.008 ^a^	0.014 ± 0.004 ^b^	0.03 ± 0.008 ^a^

Note: Data are shown as means ± SD. NG = Normal feeding group; SG = Starvation group; SBG = Starvation with BHBA infusion. ^a,b,c^ Values in the same row followed by different superscript letters are significantly different (*p* < 0.05).

## Supplementary Materials

The following are available online at https://www.mdpi.com/2076-2615/10/1/136/s1, Figure S1: The pictures of Western Bloting.
