# Supplementary figures and images for: Lipid Catabolism in Starved Yak Is Inhibited by Intravenous Infusion of β-Hydroxybutyrate
Source: Animals (Basel). 2020 Jan 15;10(1):136. doi: 10.3390/ani10010136 (PMC7022817; doi:10.3390/ani10010136)

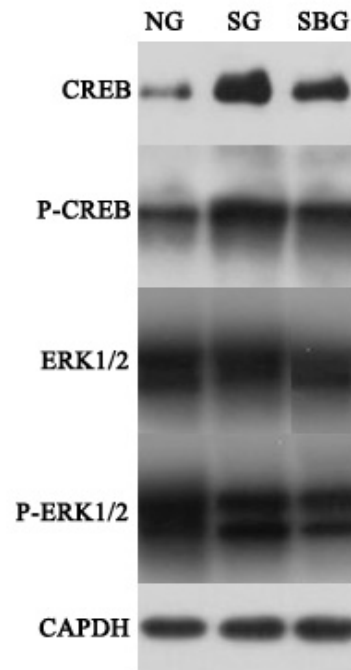

**Supplementary Figure S1.** The pictures of Western Blotting.

Supplement: Supplementary file 1 [file animals-10-00136-s001.pdf]
